# Botulinum toxin type A for genitofemoral neuralgia: A case report

**DOI:** 10.3389/fneur.2023.1228098

**Published:** 2023-07-03

**Authors:** Yan Tereshko, Belgrado Enrico, Lettieri Christian, Simone Dal Bello, Giovanni Merlino, Gian Luigi Gigli, Mariarosaria Valente

**Affiliations:** ^1^Clinical Neurology Unit, Udine University Hospital, Udine, Italy; ^2^Neurology Unit, Udine University Hospital, Udine, Italy; ^3^Department of Medicine (DAME), University of Udine, Udine, Italy

**Keywords:** botulinum toxin type A, genitofemoral nerve, pain, neuralgia, genitofemoral neuralgia

## Abstract

Genitofemoral neuralgia is an uncommon pain disorder that could be resistant to conventional treatment. A 78-year-old woman with refractory right genitofemoral neuralgia was treated with BoNT/A subcutaneous injections; the treatment was performed three times with significant pain improvement, although temporary, and without adverse events. BoNT/A may be a promising alternative intervention in the setting of genitofemoral neuralgia refractory to oral and/or topical treatment.

## 1. Introduction

The *genitofemoral nerve* (GFN) is a mainly sensory nerve (with a minor motor component innervating the cremaster muscle in males) that arises from the ventral rami of L1 and L2 of the lumbar plexus, within the psoas muscle. At the level of L3-L4, the GFN emerges from the psoas muscle and descends anteriorly along its fascia and passes posterior to the ureter and proximal to the inguinal ligament, finally bifurcating into the genital and femoral branches (1, 2). The femoral branch courses with the external iliac artery and posteriorly to the inguinal ligament; it pierces the *fascia lata* and travels into the femoral sheath, lateral to the femoral artery (1, 2). The femoral branch is a sensory nerve that conveys sensibility from the cutis of the anterior-upper thigh. The genital branch courses towards the deep inguinal ring and travels in the inguinal canal, posteromedially to the spermatic cord, to the base of the scrotum; in males, it gives motor innervation to the cremaster muscle, within the inguinal canal, and provides sensory innervation to the spermatic cord, scrotum, and upper-medial thigh in males. In females, it travels along the round ligament and innervates the *mons pubis* and *labia majora* (1, 2). Genitofemoral neuralgia is an uncommon disorder characterized by persistent or intermittent burning pain localized in the inguinal region, anteromedial upper thigh, and lateral scrotum/major labia (1, 3). Pain is relieved by assuming a bent-over position during standing while thigh extension, walking or Valsalva maneuvers exacerbate the pain (1, 2). Moreover, palpation over the pubic tubercle evokes pain and concomitant paresthesia and allodynia are common. Differential diagnosis with the ilioinguinal nerve could be challenging but clinicians suggest sequential diagnostic blocks to identify the pain generator (4). The most common cause of genitofemoral neuralgia is iatrogenic, following surgery in the inguinal region; other etiologies include traumas in the inguinal/femoral region (pubic ramus fracture and pubic symphysis irritation), indirect or direct nerve irritation, and idiopathic forms (1). Tricyclic antidepressants, lidocaine patches, gabapentin, pregabalin, and opioids are frequently used as main oral therapies (1, 2). Selective anesthetic block of the genitofemoral nerve could be effective, although rarely a permanents solution; other therapeutic approaches include radiofrequency ablation, cryoablation, and surgical intervention such as neurectomy. Botulinum toxin type A (BoNT/A) is an effective and safe treatment in neuralgias and neuropathic pain (5, 6); however, the use of BoNT/A in the setting of genitofemoral neuralgia has not been previously reported. Here we describe a woman with genitofemoral neuralgia successfully treated with subcutaneous injections of BoNT/A.

## 2. Case report

In October 2021, a 78-year-old woman came to our tertiary outpatient clinic with the complaint of persistent inguinal pain of severe burning quality (NRS 9/10). Her medical history comprehended osteoporosis, melanoma treated in 2005, and anxious-depressive disorder. She was taking pregabalin 150 mg BID, duloxetine 60 mg QD, clonazepam 30 mg QD, trazodone 75 mg QD, lidocaine patch, and paracetamol/codeine 500/30 mg TID. The pain in the inguinal region started in 2013 after a trauma with multiple fractures involving her right ilio-pubic branch and sacrum; at the beginning, the pain was intermittent, shooting and stabbing in quality, involving the inguinal region, and irradiated towards the ipsilateral labium majus and right upper-medial thigh, and exacerbated by hip extension. In the same year, she performed a selective anesthetic blockade of the right ilio-inguinal nerve without pain relief and a diagnosis while the anesthetic paravertebral block of L1 and L2 determined complete pain relief; a diagnosis of genitofemoral neuralgia was made. Through the years, the pain became gradually persistent with burning quality while pain medications became gradually ineffective. When she came to our attention, she was unable to walk due to the shooting pain induced by her right hip extension while palpation over the pubic tubercle and inguinal region exacerbated the pain; the affected area presented also dynamic mechanical allodynia and tingling paresthesia. She refused surgical therapy, cryoablation, or radiofrequency ablation. Since BoNT/A is an effective and safe therapy in different neuralgias (5, 6), we proposed BoNT/A subcutaneous injections in the territory innervated by the right genitofemoral nerve, which the patient accepted despite the off-label nature of the treatment. During the treatment period, her therapy remained unchanged.

**Figure 1:**
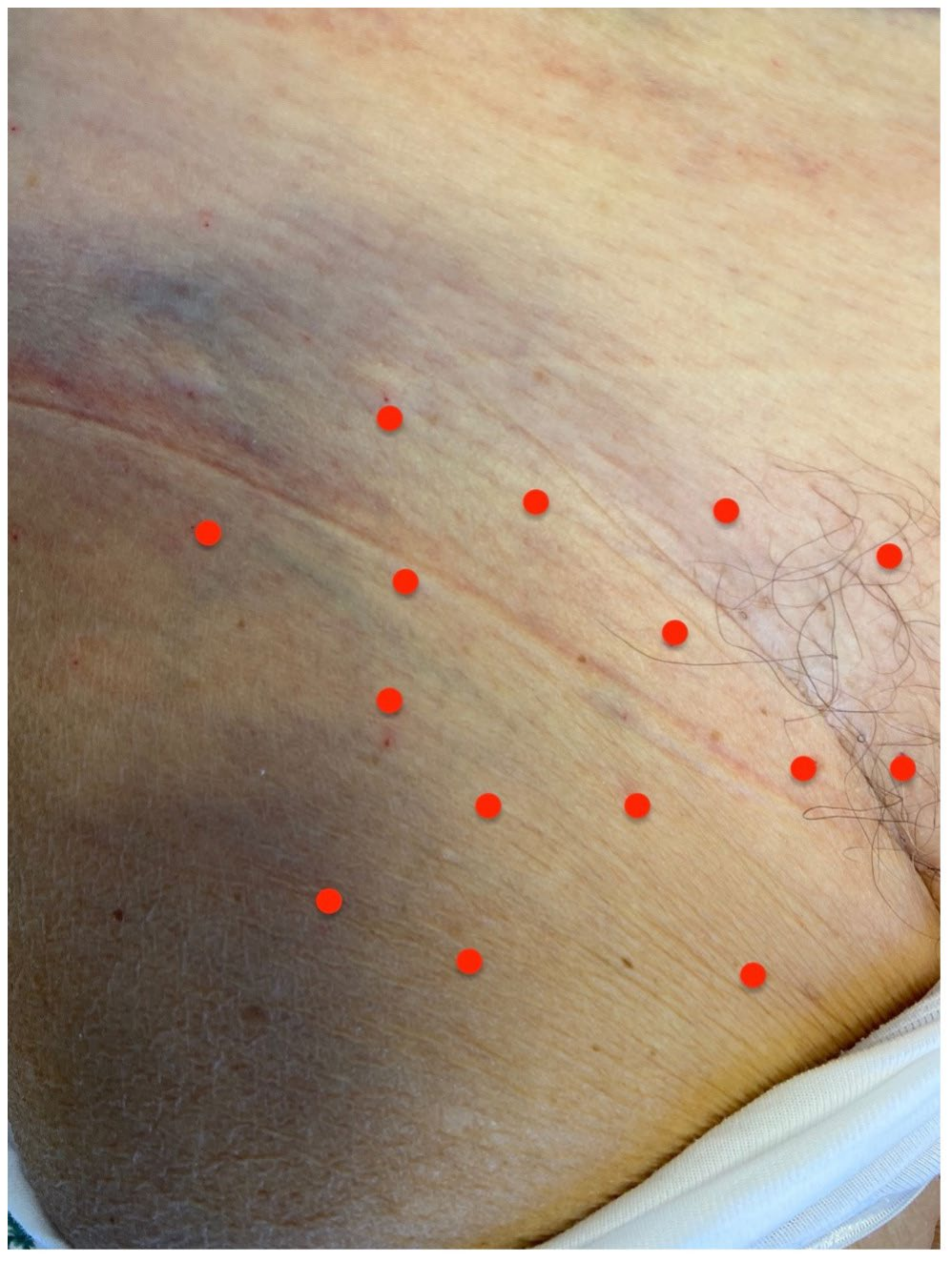
Shows the details of the first treatment with BoNT/A in our patients. The red dots represent the sites of injection (5 U per site, total dose 75 U).

The patient gave her written consent to perform the treatment with BoNT/A. She also gave her written consent to publish her images in this article. For the treatment with BoNT/A, we reconstituted a 100 U vial of onabotulinumtoxinA (BOTOX^®^) with 2 mL of sodium chloride 0.9% achieving a 50 U/mL dilution; in the third session we reconstituted two 100 U vials of onabotulinumtoxinA in the same manner. In the first round of injections, the total dose was 75 U while in the second and third sessions the total dose was 100 U and 150 U, respectively. The subcutaneous injections were performed with a 27-gage x 12.5 mm needle; we pinched the skin surrounding each injection site to separate the cutaneous layer from the muscular layer. The injections were performed with an injection angle of 30–45°. We applied topical 5% lidocaine crème for local anesthesia to reduce the injections pain. We assessed pain intensity with the Numeric Rating Scale (NRS) (7), Brief Pain Inventory scale (BPI) (8) at the baseline and 1 month and 10 days after each session. The NRS is a score to assess the pain severity using a numeric score that ranges from 0 (no pain) to 10 (worst pain) (7). The BPI is an assessment tool that provides information on the pain intensity and interference with daily activities (8, 9). We calculated the following subscales of the BPI: the BPI pain severity score and the BPI interference score. Patients’ Global Impression of Change scale (PGIC) (10) was assessed 1 month and 10 days after each session to determine the level of satisfaction of the patient with the changes in pain severity related to the treatment. The PGIC is based on a seven-point scale: no significant change is a 1–4 response while a favorable change is a 5–7 response. The patient was instructed to note the onset of action of BoNT/A, the duration of the benefit, and the adverse events. The first round of injections was performed in December 2021. The total dose for the first round of injections was 75 U (5 U per site); the injection pain was well tolerated by the patient (Figure 1).

The patient described initial pain relief 10 days after the treatment; one month and 10 days later, her pain reduced significantly (NRS 4/10), and she was able to walk. During the third month, the pain gradually returned to baseline. We performed the second round of injections in June 2022 with a total dose of 100 U of BoNT/A; in this case, the pain improved gradually 13 days after the BoNT/A subcutaneous injections and reached a maximum effect a month after (NRS 3/10). The duration of the second treatment was 3 months. The third BoNT/A injections session of BoNT/A was performed in November 2022 with a total dose of 150 U (5 U per site); pain improved gradually 11 days after the treatment and reached its maximum effect after 1 month; in this case, there was almost complete pain relief (NRS 1/10) for 4 months and then a gradual return to the baseline. There were no adverse events reported. The details of each treatment with the BPI, NRS and PGIC scores at the baseline and 1 month and 10 days after each treatment session are shown in Table 1. The patient reported being satisfied with the effect of BoNT/A on pain; she was able to perform most of the daily activities and her quality of life improved very much. The only downside of the therapy, as the patient reported, was that the effect was temporary and not permanent. The fourth round of BoNT/A injections has been delayed due to other medical complications.

**Table 1:** Shows the details of BoNT/A treatment of the patient; each BoNT/A treatment was able to temporarily reduce pain as assessed by the NRS, BPI, and PGIC.

BoNT/A treatment and dose	Assesment	NRS	BPI pain severity score	BPI interference score	PGIC	Onset BoNT/A	Duration	Adverse events
1st 75 U	T0	9/10	7.25/10	8.14/10	6	10 days	3 months	None
	T1	4/10	5/10	5.29/10				
2nd 100 U	T0	8/10	7/10	7.71/10	6	13 days	3 months	None
	T1	3/10	4/10	4.14/10				
3rd 150 U	T0	8/10	7.25/10	7.85/10	7	11 days	4 months	None
	T1	1/10	0.75/10	1.71/10				

T0, baseline pain assessment at the time of the BoNT/A injections; T1, pain assessment 1 month and 1 week after the BoNT/A treatment; BPI, Brief pain inventory score; PGIC, patient’s global impression of change; NRS, numerical rating scale.

## 3. Discussion

To our knowledge, this is the first report of genitofemoral neuralgia treated with BoNT/A. The first line of therapy in genitofemoral neuralgia are tricyclic antidepressants (amitriptyline) and/or lidocaine patches; if the first line is ineffective or insufficient, clinicians suggest the use of oral opioids, gabapentin, pregabalin, SSRI, SNRI, and NMDA antagonists. Other non-surgical approaches comprehend selective anesthetic nerve blocks, but they typically do not represent a permanent solution while radiofrequency ablation and cryoablation could be more effective in the long term (11–14). Radiofrequency ablation has shown to induce neurolysis with minimal risk to form a neuroma; this technique has proven to be effective to achieve pain relief although this may require some time and its effect may last temporarily (15). Cryoablation induces the lysis of the myelin sheath and axon of the nerve with a higher probability of the maintenance of the integrity of the perineurium and epineurium that facilitate the nerve regeneration and avoid neuroma formation; moreover, its effect antalgic effect tends to appear earlier when compared with radiofrequency ablation technique (16). Surgical therapy is suggested when the previous approaches were ineffective; in this case, genitofemoral neurectomy or triple neurectomy of all the three inguinal nerves (ilioinguinal and iliohypogastric nerves) could lead to complete pain relief (17, 18). The inevitable complications of neurectomy are the permanent anesthesia over the skin innervated by the genitofemoral nerve (and other nerves when triple neurectomy is performed) and the loss of the cremasteric reflex (1, 17, 18). Our patient was refractory to medical therapy and refused to undergo surgical treatment or ablation with radiofrequency and cryoablation. BoNT/A subcutaneous injections were performed three times with significant pain relief regarding the genitofemoral neuralgia: the first two treatments were able to significantly reduce pain while the third one was able to achieve almost complete pain remission, although for only 4 months. The duration and the efficacy could be dose-related: with higher doses, the duration and the efficacy seem to increase. Moreover, the treatment was well tolerated, and no adverse events were reported. The oral and local antalgic therapy was not altered to determine the efficacy of the treatment. BoNT/A has proven to be effective in trigeminal neuralgia, post-herpetic neuralgia, and diabetic neuropathy with a level A of evidence (19), and its mechanisms of action on neuropathic pain are several. A recent review on the use of botulinum toxin in the setting of neuropathic pain has shown that the duration and the efficacy of the antalgic effect were correlated with the dose and the sites of the injections with the exception of trigeminal neuralgia (20). The overall duration of BoNT/A was up to 3 months in trigeminal neuralgia, occipital neuralgia, Chronic regional pain syndrome, chronic pelvic pain syndrome, and auriculotemporal neuralgia (21–24) while in post-herpetic neuralgia the duration was up to 3 months (20, 25, 26). BoNT/A has an affinity for TRPV1 sensory afferents of nociceptive fibers (27, 28) and seems to reach the central nervous system through retrograde axonal and trans-synaptic transport (29–31). BoNT/A modulates neuropathic pain through the interference with the expression of TRPV1 on the plasma membrane of sensory fibers, dorsal root ganglia, and neurons in the central nervous system (29, 32). Moreover, BoNT/A inhibits the release of CGRP and substance P in both the peripheral and central nervous system, interfering with the central and peripheral sensitization mechanisms (33–36), and enhances the inhibitory opioid and GABA systems located in the dorsal horn and brainstem (37, 38). It also attenuates microglia activation (39, 40). Moreover, some studies have shown that low doses of BoNT/A are unable to spread to the central nervous system (39, 41); this could explain the heterogeneity of responses to botulinum toxin in most reports regarding neuropathic pain (20). In our case report, the duration of BoNT/A was comparable with other cases of neuropathic pain (20). The dose of botulinum toxin necessary to obtain a significant pain reduction has yet to be defined with larger studies and should be tailored on the patient since lower doses may determine an unsatisfactory response. Since genitofemoral neuralgia has neuropathic mechanisms, BoNT/A could effectively treat cases refractory to oral and topical therapies. Our patient denied adverse events and the literature describe BoNT/A as a safe therapeutic option with minimal complications. A placebo effect is unlikely in our case report since the effect of BoNT/A was temporary and had a delayed onset of action. However, we cannot exclude a concomitant possible placebo effect of unknown degree.

## 4. Conclusion

BoNT/A could be a safe and effective therapy for refractory cases of genitofemoral neuralgia. However, larger studies with a placebo-control design are needed to confirm its efficacy and the appropriate dose. We hope our case report will prompt other clinicians to use BoNT/A in this setting.

## Data availability statement

The original contributions presented in the study are included in the article/supplementary material, further inquiries can be directed to the corresponding author.

## Ethics statement

Ethical review and approval was not required for the study on human participants in accordance with the local legislation and institutional requirements. The patients/participants provided their written informed consent to participate in this study.

## Author contributions

YT: conceptualization, investigation, data curation, visualization, and writing – original draft. BE: conceptualization, supervision, writing – review and editing, resources, project administration, and supervision. LC: writing – review and editing and visualization. SD, GM, and GG: writing – review & editing. MV: writing – review and editing, project administration, and supervision. All authors contributed to the article and approved the submitted version.

## Conflict of interest

The authors declare that the research was conducted in the absence of any commercial or financial relationships that could be construed as a potential conflict of interest.

## Publisher’s note

All claims expressed in this article are solely those of the authors and do not necessarily represent those of their affiliated organizations, or those of the publisher, the editors and the reviewers. Any product that may be evaluated in this article, or claim that may be made by its manufacturer, is not guaranteed or endorsed by the publisher.

## Abbreviations

GFN: genitofemoral nerve
BoNT/A: Botulinum toxin type A
TRPV1: transient receptor potential vanilloid 1.

## References

[1] Cesmebasi A Yadav A Gielecki J Tubbs RS Loukas M. Genitofemoral neuralgia: a review. Clin Anat. (2015) 28:128–35. doi: 10.1002/ca.22481, PMID: 25377757

[2] Vanetti TK Luba ATR Assis FD de Oliveira CA. Genitofemoral Nerve Entrapment: Pelvic In: Trescot AM, editor. Peripheral Nerve Entrapments. Cham: Springer (2016).

[3] Harms BA DeHaas DRJ Starling JR. Diagnosis and management of genitofemoral neuralgia. Arch Surg. (1984) 119:339–41. doi: 10.1001/archsurg.1984.01390150071017 6696629

[4] Kale A Aytuluk HG Cam I Basol G Sunnetci B. Selective spinal nerve block in Ilioinguinal, Iliohypogastric and genitofemoral neuralgia. Turk Neurosurg. (2019) 29:237–530. doi: 10.5137/1019-5149.JTN.23990-18.1 30829381

[5] Datta Gupta A Edwards S Smith J Snow J Visvanathan R Tucker G. A systematic review and Meta-analysis of efficacy of botulinum toxin a for neuropathic pain. Toxins (Basel). (2022) 14:36. doi: 10.3390/toxins14010036, PMID: 35051013 PMC8780616

[6] Oh H-M Chung ME. Botulinum toxin for neuropathic pain: a review of the literature. Toxins (Basel). (2015) 7:3127–54. doi: 10.3390/toxins7083127, PMID: 26287242 PMC4549742

[7] Thong ISK Jensen MP Miró J Tan G. The validity of pain intensity measures: What do the NRS, VAS, VRS, and FPS-R measure? Scand J Pain. (2018) 18:99–107. doi: 10.1515/sjpain-2018-0012 29794282

[8] Im DD Jambaulikar GD Kikut A Gale J Weiner SG. Brief pain inventory–short form: a new method for assessing pain in the emergency department. Pain Med. (2020) 21:3263–9. doi: 10.1093/pm/pnaa269 32918473

[9] Kumar SP. Utilization of brief pain inventory as an assessment tool for pain in patients with cancer: a focused review. Indian J Palliat Care. (2011) 17:108–15. doi: 10.4103/0973-1075.84531, PMID: 21976850 PMC3183599

[10] Hurst H Bolton J. Assessing the clinical significance of change scores recorded on subjective outcome measures. J Manip Physiol Ther. (2004) 27:26–35. doi: 10.1016/j.jmpt.2003.11.003 14739871

[11] Falzone U Santonocito C Zanghì MG Rinzivillo N Provenzano D Sapienza E. Neuropathic inguinal pain: neurectomy associated with open prosthetic hernioplasty for the prevention of post-operative pain. Ann Ital Chir. (2022) 93:377–84. PMID: 36155937

[12] Fanelli RD DiSiena MR Lui FY Gersin KS. Cryoanalgesic ablation for the treatment of chronic postherniorrhaphy neuropathic pain. Surg Endosc. (2003) 17:196–200. doi: 10.1007/s00464-002-8840-8, PMID: 12457217

[13] Trescot AM. Cryoanalgesia in interventional pain management. Pain Physician. (2003) 6:345–60. doi: 10.36076/ppj.2003/6/345, PMID: 16880882

[14] Tao J Huang B Tang J Luo G Zhu J He Q. Comparison of efficacy and safety of lumbar sympathetic radiofrequency Thermocoagulation versus chemical lumbar Sympathectomy in the treatment of cold hypersensitivity in the hands and feet: a retrospective study. Pain Physician. (2022) 25:E357–64. PMID: 35322991

[15] Parris D Fischbein N Mackey S Carroll I. A novel CT-guided transpsoas approach to diagnostic genitofemoral nerve block and ablation. Pain Med. (2010) 11:785–9. doi: 10.1111/j.1526-4637.2010.00835.x, PMID: 20546515 PMC2913614

[16] Campos NA Chiles JH Plunkett AR. Ultrasound-guided cryoablation of genitofemoral nerve for chronic inguinal pain. Pain Physician. (2009) 12:997–1000. doi: 10.36076/ppj.2009/12/997, PMID: 19935984

[17] Murovic JA Kim DH Tiel RL Kline DG. Surgical management of 10 genitofemoral neuralgias at the Louisiana State University health sciences center. Neurosurgery. (2005) 56:298–303. doi: 10.1227/01.neu.0000148000.04592.e1, PMID: 15670378

[18] Acar F Ozdemir M Bayrakli F Cirak B Coskun E Burchiel K. Management of medically intractable genitofemoral and ilioingunal neuralgia. Turk Neurosurg. (2013) 23:753–7. doi: 10.5137/1019-5149.JTN.7754-12.0 24310458

[19] Park J Park HJ. Botulinum toxin for the treatment of neuropathic pain. Toxins (Basel). (2017) 9:260. doi: 10.3390/toxins9090260, PMID: 28837075 PMC5618193

[20] Egeo G Fofi L Barbanti P. Botulinum neurotoxin for the treatment of neuropathic pain. Front Neurol. (2020) 11:716. doi: 10.3389/fneur.2020.00716, PMID: 32849195 PMC7431775

[21] Taylor M Silva S Cottrell C. Botulinum toxin type-a (BOTOX) in the treatment of occipital neuralgia: a pilot study. Headache. (2008) 48:1476–81. doi: 10.1111/j.1526-4610.2008.01089.x 19076646

[22] Morra ME Elgebaly A Elmaraezy A Khalil AM Altibi AMA Vu TL-H. Therapeutic efficacy and safety of botulinum toxin a therapy in trigeminal neuralgia: a systematic review and meta-analysis of randomized controlled trials. J Headache Pain. (2016) 17:63. doi: 10.1186/s10194-016-0651-8, PMID: 27377706 PMC4932020

[23] Tereshko Y Belgrado E Lettieri C Gigli GL Valente M. Botulinum toxin type A for the treatment of Auriculotemporal neuralgia-a case series. Toxins (Basel). (2023) 15:274. doi: 10.3390/toxins15040274, PMID: 37104212 PMC10141838

[24] Tereshko Y Dalla Torre C Lettieri C Belgrado E Gigli GL Valente M. Subcutaneous BoNT/a injection for intractable pain and disability in complex regional pain syndrome: a case report. Toxins (Basel). (2022) 14:411. doi: 10.3390/toxins14060411, PMID: 35737072 PMC9227913

[25] Attal N de Andrade DC Adam F Ranoux D Teixeira MJ Galhardoni R. Safety and efficacy of repeated injections of botulinum toxin a in peripheral neuropathic pain (BOTNEP): a randomised, double-blind, placebo-controlled trial. Lancet Neurol. (2016) 15:555–65. doi: 10.1016/S1474-4422(16)00017-X, PMID: 26947719

[26] Gottsch HP Miller JL Yang CC Berger RE. A pilot study of botulinum toxin for interstitial cystitis/painful bladder syndrome. Neurourol Urodyn. (2011) 30:93–6. doi: 10.1002/nau.20946, PMID: 20589903

[27] Matak I Rossetto O Lacković Z. Botulinum toxin type A selectivity for certain types of pain is associated with capsaicin-sensitive neurons. Pain. (2014) 155:1516–26. doi: 10.1016/j.pain.2014.04.027, PMID: 24793910

[28] Yiangou Y Anand U Otto WR Sinisi M Fox M Birch R. Increased levels of SV2A botulinum neurotoxin receptor in clinical sensory disorders and functional effects of botulinum toxins a and E in cultured human sensory neurons. J Pain Res. (2011) 4:347–55. doi: 10.2147/JPR.S25189, PMID: 22090803 PMC3215514

[29] Lacković Z Filipović B Matak I Helyes Z. Activity of botulinum toxin type A in cranial dura: implications for treatment of migraine and other headaches. Br J Pharmacol. (2016) 173:279–91. doi: 10.1111/bph.13366, PMID: 26493010 PMC5341233

[30] Marinelli S Vacca V Ricordy R Uggenti C Tata AM Luvisetto S. The analgesic effect on neuropathic pain of retrogradely transported botulinum neurotoxin a involves Schwann cells and astrocytes. PLoS One. (2012) 7:e47977. doi: 10.1371/journal.pone.0047977, PMID: 23110146 PMC3480491

[31] Ramachandran R Lam C Yaksh TL. Botulinum toxin in migraine: role of transport in trigemino-somatic and trigemino-vascular afferents. Neurobiol Dis. (2015) 79:111–22. doi: 10.1016/j.nbd.2015.04.011, PMID: 25958249 PMC4458441

[32] Shimizu T Shibata M Toriumi H Iwashita T Funakubo M Sato H. Reduction of TRPV1 expression in the trigeminal system by botulinum neurotoxin type-a. Neurobiol Dis. (2012) 48:367–78. doi: 10.1016/j.nbd.2012.07.010, PMID: 22820141

[33] McMahon HT Foran P Dolly JO Verhage M Wiegant VM Nicholls DG. Tetanus toxin and botulinum toxins type a and B inhibit glutamate, gamma-aminobutyric acid, aspartate, and met-enkephalin release from synaptosomes. Clues to the locus of action. J Biol Chem. (1992) 267:21338–43. doi: 10.1016/S0021-9258(19)36614-1, PMID: 1356988

[34] Nakov R Habermann E Hertting G Wurster S Allgaier C. Effects of botulinum a toxin on presynaptic modulation of evoked transmitter release. Eur J Pharmacol. (1989) 164:45–53. doi: 10.1016/0014-2999(89)90229-x, PMID: 2568939

[35] Durham PL Cady R Cady R. Regulation of calcitonin gene-related peptide secretion from trigeminal nerve cells by botulinum toxin type A: implications for migraine therapy. Headache. (2004) 44:33–5. doi: 10.1111/j.1526-4610.2004.04007.x, PMID: 14979881

[36] Purkiss J Welch M Doward S Foster K. Capsaicin-stimulated release of substance P from cultured dorsal root ganglion neurons: involvement of two distinct mechanisms. Biochem Pharmacol. (2000) 59:1403–6. doi: 10.1016/s0006-2952(00)00260-4, PMID: 10751549

[37] Drinovac V Bach-Rojecky L Matak I Lacković Z. Involvement of μ-opioid receptors in antinociceptive action of botulinum toxin type A. Neuropharmacology. (2013) 70:331–7. doi: 10.1016/j.neuropharm.2013.02.011, PMID: 23499661

[38] Drinovac V Bach-Rojecky L Lacković Z. Association of antinociceptive action of botulinum toxin type A with GABA-A receptor. J Neural Transm. (2014) 121:665–9. doi: 10.1007/s00702-013-1150-6, PMID: 24420081

[39] Piotrowska A Popiolek-Barczyk K Pavone F Mika J. Comparison of the expression changes after botulinum toxin type A and minocycline Administration in Lipopolysaccharide-Stimulated rat Microglial and Astroglial Cultures. Front Cell Infect Microbiol. (2017) 7:141. doi: 10.3389/fcimb.2017.00141, PMID: 28491822 PMC5405066

[40] Mika J Rojewska E Makuch W Korostynski M Luvisetto S Marinelli S. The effect of botulinum neurotoxin a on sciatic nerve injury-induced neuroimmunological changes in rat dorsal root ganglia and spinal cord. Neuroscience. (2011) 175:358–66. doi: 10.1016/j.neuroscience.2010.11.040, PMID: 21111791

[41] Filipović B Matak I Bach-Rojecky L Lacković Z. Central action of peripherally applied botulinum toxin type A on pain and dural protein extravasation in rat model of trigeminal neuropathy. PLoS One. (2012) 7:e29803. doi: 10.1371/journal.pone.0029803, PMID: 22238656 PMC3251614

